# Respiratory Diphtheria in a 16-Year-Old Who Developed Multiple Life-Threatening Complications

**DOI:** 10.1016/j.acepjo.2025.100043

**Published:** 2025-02-08

**Authors:** Thomas Boisdenghien, Juliette Genot, Mahmoud Kaabour, Axel Derwa, Sergio Rizzi, Marie Belleflamme

**Affiliations:** 1 Department of Cardiology, Cliniques universitaire de Bruxelles, ULBruxelles, Bruxelles, Belgium; 2 Department of Emergency Medicine, Cliniques universitaires Saint-Luc, UCLouvain, Bruxelles, Belgium; 3 Department of Emergency Medicine, Clinic Saint-Jean, Bruxelles, Belgium; 4 Department of Internal Medicine and Nephrology, Clinic Saint-Jean, Bruxelles, Belgium; 5 Department of Cardiology, Clinic Saint-Jean, Bruxelles, Belgium

**Keywords:** emergency, respiratory diphtheria, diphtheria management

## Abstract

Diphtheria is a contagious disease with high mortality. Although the global prevalence has been decreasing, the immigration of certain populations with suboptimal vaccination coverage has contributed to its resurgence in developed countries. We report a 16-year-old Afghan male with acute respiratory distress and life-threatening complications, including fulminant myocarditis with cardiac arrhythmias and neurologic complications. This case highlights the significance of optimal vaccination coverage, especially the vaccination status of migrants. This paper serves as a reminder of diphtheria's systemic impact and underscores the need for a multidisciplinary approach to respiratory diphtheria management. Vigilant monitoring, prompt immunoglobulin and antibiotic administration, and supportive care all play pivotal roles in enhancing patient outcomes and reducing mortality risks.

## 1 Introduction

Diphtheria is caused by *Corynebacterium diphtheriae*, a Gram-positive aerobic bacterium associated with high mortality rates (10%). Diphtheria's virulence stems from the ability to express the *tox* gene to produce diphtheroid toxin, closely tied to pseudomembrane severity, leading to severe systemic complications by inhibiting protein synthesis, leading to cell degeneration and necrosis.

The treatment relies on early immunoglobulin and antibiotic therapy, usually combining a macrolide and penicillin G.^1^

The worldwide availability of vaccines has led to a significant global decline in prevalence. In 2020, the World Health Organization (WHO) reported an 83% global vaccine coverage, contributing to a more than 90% reduction in cases between 1980 and 2000. Diphtheria is currently rare in Europe, with a mere 451 cases documented by the WHO between 2010 and 2019. However, with the increasing immigration, Europe has been witnessing a rise in sporadic diphtheria cases.^2^

Additionally, rare diseases in Europe, such as typhoid fever, leptospirosis, and viral hepatitis are reemerging. Indeed, in 2019, one-third of tuberculosis cases and just under 50% of diagnosed HIV cases in Europe were among migrants.^3^^,^^4^

## 2 Presentation of the Clinical Case

A 16-year-old Afghan male presented at the emergency department in Belgium with odynophagia and respiratory congestion. He lacked a significant past medical history, and language barriers posed challenges to communication. The patient did not exhibit dysphagia, fever, thoracic pain, or dyspnea. However, he presented with marked fatigue, periorbital dark circles, and a pale facial appearance. On admission, the vital signs included a blood pressure of 110/60 mm Hg, a heart rate of 73 bpm, and an oxygen saturation of 94%. An ear, nose, and throat specialist performed a throat examination, revealing erythema, bilateral swollen tonsils, and the presence of pseudomembranes, indicating the detachment of necrosed tonsillar tissue. The patient exhibited bilateral swollen cervical lymph nodes, creating a characteristic “bull neck” appearance.

The main differential diagnoses included streptococcal pharyngitis, infectious mononucleosis, atypical infections (eg, gonococcus, chlamydia), and severe HIV infection. Diphtheria was not initially considered among the primary differential diagnoses. However, emergency physicians were prompted to include diphtheria due to a recent case of cutaneous diphtheria in a migrant patient in the same hospital.

Complete blood count showed neutrophilic leukocytosis (25.4 × 10^3^/mm^3^), and biochemistry had elevated inflammatory markers (C-reactive protein 302 mg/dL; normal < 5 mg/dL). An electrocardiogram and a pulmonary x-ray did not reveal any abnormalities. An oropharyngeal swab and viral serologies were performed, with polymerase chain reaction results from the oropharyngeal sample confirming infection with *Corynebacterium diphtheriae* within 2 hours.

Treatment was initiated with intravenous antibiotics, including penicillin G (600,000 Units twice a day) and erythromycin (500 mg 3 times a day).

Given the patient's stable condition, he was initially hospitalized for intravenous antibiotic administration and close monitoring of his symptoms. However, he developed acute respiratory distress overnight, necessitating urgent endotracheal intubation just hours after leaving the emergency department. Antidiphtheria immunoglobulins (100,000 Units) were administered within 72 hours but were delayed in the setting of supply chain difficulties and needed to be obtained from abroad.

On the fifth day, he developed supraventricular tachycardia and became hemodynamically unstable. An intravenous loading dose of amiodarone was administered, followed by multiple unsuccessful attempts of electrical cardioversion involving 5 electric shocks. As part of the treatment, amiodarone infusion was continued, along with the administration of supportive vasopressor (norepinephrine). The results of a serum troponin test indicated elevated levels at 11,040 ng/L (normal < 5 ng/L). A cardiac ultrasound was performed and revealed a decreased ejection fraction of the left ventricle at 47%, accompanied by inferior akinesia and septal dyskinesia. The impairment of left ventricular function supported by laboratory findings, along with multiple arrhythmias, provided clinical evidence supporting a diagnosis of diphtheria myocarditis.

**Figure 1:**
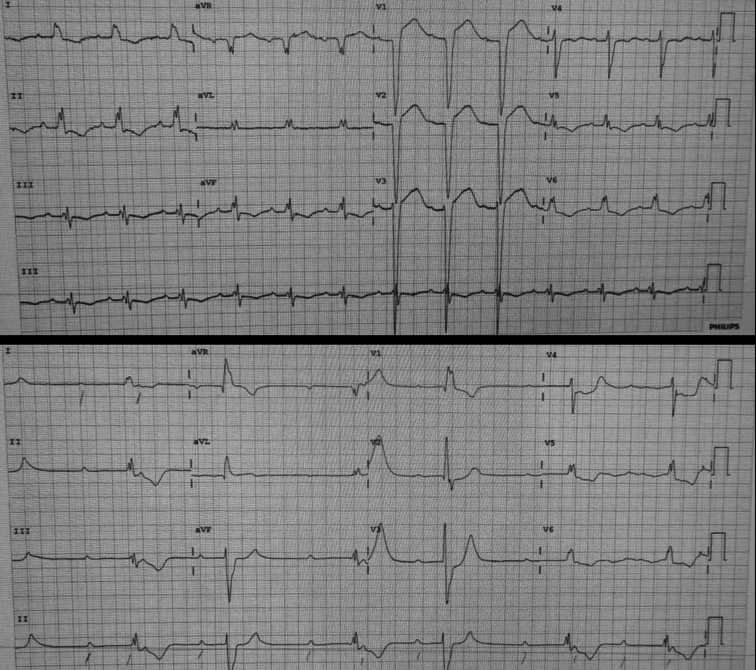
Arrhythmia in diphtheritic myocarditis. Visual representation demonstrating the temporal evolution of the patient's electrocardiogram with myocarditis. Initially, there was evidence of first-degree atrioventricular block concomitant with de novo left bundle branch block. Subsequent electrocardiograms, obtained over the course of several days, revealed a progression to complete atrioventricular block, culminating in profound bradycardia and eventual asystole.

On the seventh day, an atrioventricular block developed, progressing to complete block (Fig 1). An infusion of isoprenaline was initiated. However, by the 14th day, the patient developed severe bradycardia, which progressed to asystole, necessitating cardiopulmonary resuscitation and resulting in the return of spontaneous circulation.

A temporary transvenous pacemaker and extracorporeal membrane oxygenation (ECMO) were implemented.

The support systems were gradually reduced as the patient's condition stabilized, and biomarkers showed significant improvement. To address any underlying cardiac abnormalities, the patient received a 2-chamber permanent pacemaker.

**Figure 2:**
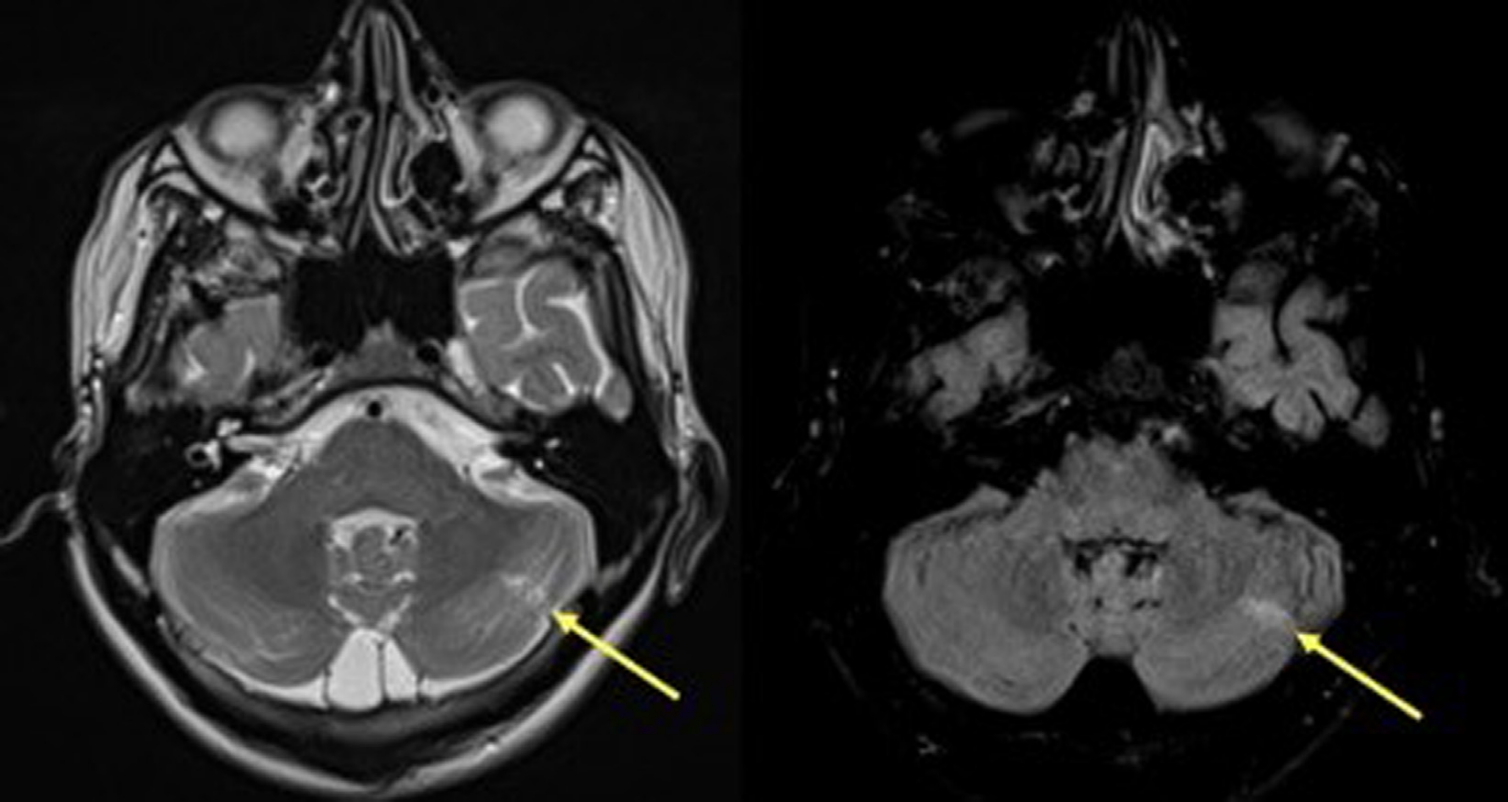
Possibility of cerebellar involvement because of diphtheria. Cerebral magnetic resonance imaging revealed 2 lacunar ischemic sequelae, one in the right posterior parietal region and the other in the left cerebellum (yellow arrows). A linear hyperintense area in T2/FLAIR, predominantly cortical, was detected in the left middle third of the cerebellum. FLAIR, fluid-attenuated inversion recovery.

Post-ECMO removal, neurologic symptoms manifested, such as left hemineglect and left paraparesis. Cerebral magnetic resonance imaging (MRI) revealed 2 lacunar ischemic areas (Fig 2). Further investigations, including electromyography, electroencephalography, and brachial plexus MRI, ruled out peripheral causes. This, combined with the brain lesions observed on MRI, suggests probable diphtheria-related central nervous system complications. However, it remains uncertain whether these findings are directly attributable to diphtheria or could be linked to other factors, such as the ECMO procedure or cardiopulmonary resuscitation.

Following his stay in intensive care and rehabilitation, the patient was discharged with severe left systolic dysfunction with an ejection fraction of 35%. A year later, he had fully recovered from neurologic symptoms but still had residual septal dyskinesia. However, his overall condition had improved, with an ejection fraction of 50%.

## 3 Discussion

With the increasing immigration, Europe has witnessed a rise in sporadic diphtheria cases.

This case underscores the need for health care professionals to consider diphtheria diagnosis, especially in those from endemic regions (ie, Central and South America, Africa, Asia, Russia, and Eastern Europe) with acute respiratory symptoms.

When evaluating upper respiratory conditions in migrant patients, it is essential to consider a broad spectrum of infectious diseases beyond the common ones, like pharyngitis or epiglottitis. Comprehensive testing, including viral and bacterial cultures of nasopharyngeal samples (targeting pathogens like adenovirus, influenza A and B, Epstein-Barr virus, *Streptococcus pneumoniae*, *Staphylococcus aureus*, *Haemophilus influenzae*, and *Corynebacterium diphtheriae*), is critical. Additionally, particular attention should be paid to the patient’s vaccination status, as incomplete immunization may predispose them to preventable yet severe infections like diphtheria.

In respiratory diphtheria, pseudomembrane invasion and neck tissue swelling lead to complications such as respiratory tract obstruction and acute respiratory distress.

When patients exhibit one of those symptoms, it is crucial to rapidly secure the airways. Opinions differ on choosing endotracheal intubation or emergency tracheostomy, with experts recommending the approach based on physician expertise and the clinical context.

In our case, no cervical computed tomography scan was performed as an ENT specialist conducted a reassuring fibroscopy quickly after admission and revealed no tonsillar abscess.

Furthermore, despite signs like a bull neck, prominent pseudo membranes, and mild desaturation, we initially opted against immediate intubation due to the patient's stability. In hindsight, considering the rapid deterioration that followed, earlier intubation would have been warranted to preempt the acute respiratory distress that ultimately necessitated urgent intervention.

The virulence of diphtheria is primarily attributed to the presence of diphtheria toxin, which is closely correlated with the severity of the pseudomembranes. It explains why respiratory diphtheria can lead to significant complications across multiple body systems. It is crucial to neutralize the toxin with antitoxin before it binds to cells. The treatment lies in the early administration of the immunoglobulin to reduce the risk of toxin dissemination and antibiotic therapy, usually combining a macrolide, such as an erythromycin and penicillin G.^1^^,^^5^ In this case, the delay with immunoglobulins due to the lack of availability of the antitoxin in Belgium (imported from the Netherlands) was deleterious, and earlier administration could have prevented cardiac and neurologic complications. Antibiotic treatment targets only respiratory diphtheria and does not neutralize the toxins responsible for these complications.

Cardiac complications affect 10% to 20% of severe respiratory diphtheria cases, with mortality rates of 20% to 70%.^6^^,^^7^ Myocarditis appears within the first 2 weeks and induces arrhythmias, conduction abnormalities, repolarization abnormalities, and heart failure.^6^^,^^8^ These rhythm disturbances are primarily attributed to significant inflammation of the sinoatrial and atrioventricular nodes.^9^

Our case demonstrated supraventricular tachycardia evolving into complete atrioventricular block. These observed rhythm abnormalities are consistent with the severity of diphtheria-associated myocarditis as described in the literature and contribute to the high mortality rate.^6^^,^^7^^,^^9^

The treatment for acute myocarditis remains hemodynamic support. We were able to stabilize the patient with veno-arterial ECMO and decided to implant a temporary pacemaker despite differing opinions in the literature. In a study by Stockins et al,^10^ no survival benefit was observed in a series of 11 patients affected by a complete atrioventricular block who received a temporary pacemaker. Initially, there was hesitation regarding the implementation of an external pacemaker due to its associated risks, such as infections, thrombosis, and myocardial perforation occurring in 0.5% to 3% of cases.^11^ However, a recent study conducted in Vietnam with 42 patients showed a mortality reduction of over 70% when a temporary pacemaker was implanted.^12^

In our case, the ventricular dysfunction probably resulted from tachycardia-induced cardiomyopathy and inflammation of the myocardium. Restoring adequate cardiac frequency through pacemaker intervention permitted for hemodynamic stabilization.

By implanting the temporary pacemaker, we were able to wean the patient off ECMO support in less than a week. Considering the persistence of complete atrioventricular block, we decided to convert this to a permanent pacemaker.

Neurologic complications in diphtheria, linked to toxin absorption by Schwann cells, are rare, occurring in only 5% of cases, and typically manifest within the month preceding the onset of severe respiratory symptoms.^1^^,^^8^ They are sparsely documented in the literature. Manifestations include palatal and bulbar palsy progressing to polyneuropathy, with isolated reports of central nervous system type of diphtheritic encephalitis.^13^^,^^14^

Our case ruled out peripheral involvement by conducting investigations with neurologists, but showed cerebral abnormalities. However, the cerebral MRI revealed a hyperintense area, which was also observed in another case report describing diphtheritic encephalitis.^13^ Limited literature and our radiologists' unfamiliarity with this presentation make it difficult to attribute the findings to diphtheria or ECMO complications.

## 4 Conclusion

This case highlights the critical importance of early recognition and aggressive management of respiratory diphtheria. Prompt actions, including emergency intubation, immediate immunoglobulin and antibiotic treatment, and addressing complications, underscore the need for a multidisciplinary approach to enhance patient outcomes and reduce mortality risks, emphasizing the necessity for improved knowledge and resources.

## Funding and Support

Supported by the Nephrology Department, Clinic Saint-Jean, Bruxelles, Belgium.

## Conflict of Interest

All authors have affirmed they have no conflicts of interest to declare.
